# Cost-minimisation model of magnetic resonance-guided focussed ultrasound therapy compared to unilateral deep brain stimulation for essential tremor treatment in Japan

**DOI:** 10.1371/journal.pone.0219929

**Published:** 2019-07-17

**Authors:** Ataru Igarashi, Midori Tanaka, Keiichi Abe, Lance Richard, Vivian Peirce, Kazumichi Yamada

**Affiliations:** 1 Health Economics and Outcomes Research, University of Tokyo, Tokyo, Japan; 2 Department of Pharmaceutical Sciences, University of Tokyo, Tokyo, Japan; 3 Department of Neurosurgery, Tokyo Women’s Medical University, Tokyo, Japan; 4 INSIGHTEC Ltd, Tirat Carmel, Israel; 5 Costello Medical Consulting, Cambridge, United Kingdom; 6 Department of Neurosurgery, Kumamoto University, Kumamoto, Japan; University of California Los Angeles, UNITED STATES

## Abstract

**Objective:**

To investigate the cost differences between magnetic resonance-guided focussed ultrasound (MRgFUS) and unilateral deep brain stimulation (DBS) for the treatment of medication-refractory essential tremor (ET) in Japan using a cost-minimisation model.

**Methods:**

A cost-minimisation model estimated total costs for MRgFUS and unilateral DBS by summing the pre-procedure, procedure, and post-procedure costs over a 12-month time horizon, using data from published sources and expert clinical opinion. The model base case considered medical costs from fee-for-service tariffs. Scenario analyses investigated the use of Diagnosis Procedure Combination tariffs, a diagnosis-related group-based fixed-payment system, and the addition of healthcare professional labour costs healthcare professionals using tariffs from the Japanese Health Insurance Federation for Surgery. One-way sensitivity analyses altered costs associated with tremor recurrence after MRgFUS, the extraction rate following unilateral DBS, the length of hospitalisation for unilateral DBS and the procedure duration for MRgFUS. The impact of uncertainty in model parameters on the model results was further explored using probabilistic sensitivity analysis.

**Results:**

Compared to unilateral DBS, MRgFUS was cost saving in the base case and Diagnosis Procedure Combination cost scenario, with total savings of JPY400,380 and JPY414,691, respectively. The majority of savings were accrued at the procedural stage. Including labour costs further increased the cost differences between MRgFUS and unilateral DBS. Cost savings were maintained in each sensitivity analysis and the probabilistic sensitivity analysis, demonstrating that the model results are highly robust.

**Conclusions:**

In the Japanese healthcare setting, MRgFUS could be a cost saving option versus unilateral DBS for treating medication-refractory ET. The model results may even be conservative, as the cost of multiple follow-ups for unilateral DBS and treatment costs for adverse events associated with each procedure were not included. This model is also consistent with the results of other economic analyses of MRgFUS versus DBS in various settings worldwide.

## Introduction

Essential tremor (ET) is the most dominant form of movement disorder worldwide, with a prevalence of 4.6% in individuals aged ≥65 years [1]. ET is a chronic and progressive neurological disease that commonly manifests as bilateral tremors in the upper limbs, and is associated with considerable physical and psychosocial burden [2, 3]. Many patients struggle with everyday tasks such as writing, dressing and/or eating and have a diminished health-related quality of life (HRQoL) [3, 4]. The condition may also result in decreased productivity, and in premature retirement for those suffering from its symptoms [5].

While many patients respond well to first-line oral medications and achieve a reduction in tremulous symptoms, approximately 30–50% of ET patients are medication-refractory [6, 7]. This leaves a substantial proportion suffering from poor dexterity and a diminished HRQoL [4, 8]. Alternative treatment options for these medication-refractory ET patients include procedural interventions that ablate or stimulate the ventral intermediate nucleus (VIM) of the thalamus, such as radiofrequency thalamotomy (RFT), stereotactic radiosurgery (SRS), deep brain stimulation (DBS) and magnetic resonance-guided focussed ultrasound (MRgFUS) [7, 9]. In Japan, RFT and SRS are not frequently performed: RFT has largely been replaced by less invasive or reversible procedures, and SRS is not currently reimbursed by national insurance. However, DBS is commonly performed, and MRgFUS is also available in many hospitals following its regulatory approval by the Ministry of Health, Labour and Welfare in Japan in 2016 [10].

Upon its introduction in 1993, DBS rapidly became the standard surgical treatment for medication-refractory movement disorders, including ET [9, 11, 12]. Numerous studies evaluating DBS have reported immediate tremor improvements with long-term follow-up showing a 40–80% reduction in tremor symptoms and a corresponding improvement in HRQoL [13]. DBS can be performed unilaterally or bilaterally and is a reversible intervention, offering physicians the option to adjust stimulation parameters to balance tremor control with the risk for side effects [14–16]. The latter provides a level of safety that led to its preferred use over irreversible techniques such as RFT. However, DBS is an invasive procedure and as a result, carries a risk of surgical complications and requires a lengthy hospital stay [17, 18]. Infections, device-related complications or battery depletion may also occur, necessitating extraction or revision operations; such additional procedures incur further direct medical costs after the initial procedure [8, 13, 19–22].

In contrast, MRgFUS is a minimally invasive technique performed unilaterally that ablates the VIM by combining focussed ultrasound ablation with magnetic resonance thermometry, to permit the monitoring of treatment via thermal feedback in real-time [9]. Unilateral MRgFUS is performed without the need for an operative procedure or associated general anaesthesia, so post-procedure recovery requires only one night as an inpatient in hospital [23]. Furthermore, a large-scale, randomised controlled trial (RCT) in patients with medication-refractory ET demonstrated significant and immediate improvement in hand tremors post-MRgFUS versus a sham procedure [7]. This relief was seen to be durable, with symptom relief significantly maintained at both two- and four-year follow-up, providing evidence for long-term efficacy of this procedure [24, 25]. In an analysis of five individual studies of MRgFUS, procedure-related serious adverse events (AEs) were very infrequent (1.6%), without intracerebral haemorrhages or infections. In addition, reported AEs were largely transient and were commonly rated as mild (79%) and rarely severe (1%) [26]. This is also supported by four-year follow-up data for MRgFUS, where no permanent adverse effects were reported, and there were no newly developed AEs during the follow-up period [25].

Given the recent regulatory approval of MRgFUS by the Ministry of Health, Labour and Welfare in Japan and its advantages over DBS as described above, there is a potential for MRgFUS to become an alternative treatment option to DBS in Japanese clinical practice [10]. Although the regulatory approval of MRgFUS does not specify the sidedness of the procedure [10], MRgFUS is only used unilaterally in Japan, based on the input of clinical experts. Therefore, unilateral DBS represents the appropriate comparator for MRgFUS in cost analyses, given its analogous mechanism of action to MRgFUS in targeting one side of the brain only. However, there are few economic evaluations of MRgFUS in medication-refractory ET, and none thus far that compare MRgFUS to unilateral DBS in the Japanese healthcare setting [27–29]. Consequently, there is a need for a comparison of the costs associated with MRgFUS and unilateral DBS for the treatment of medication-refractory ET in Japan.

Evidence from a small retrospective analysis and an indirect treatment comparison (ITC) of MRgFUS and unilateral DBS with 12 months follow-up shows that these procedures provide similar relief from tremor and its associated disabilities [15, 30]. Therefore, a cost-minimisation model, which assumes equal efficacy for each included procedure, can be considered an appropriate approach for comparing the costs of MRgFUS to unilateral DBS across a 12-month time horizon. The objective of conducting this economic analysis was to determine whether MRgFUS is cost saving versus unilateral DBS in the treatment of medication-refractory ET in Japan.

## Methods

### Population

The economic evaluation considered simulated patients with medication-refractory ET treated with either MRgFUS or unilateral DBS in Japan.

### Design and structure of the economic model

Based on an assumption of equal efficacy, a cost-minimisation model from a Japanese healthcare payers’ perspective was used to compare MRgFUS to unilateral DBS, capturing only direct healthcare costs [15, 30–32]. All model costs were estimated using 2018 Japanese Yen (JPY) except where explicitly stated in analyses.

Modelling of simulated patients commenced prior to the MRgFUS or unilateral DBS procedure, with costs involved in the planning of, during and after the procedure considered over a time horizon of 12 months. Costs at each stage were summed to generate total costs per procedure for MRgFUS and unilateral DBS, respectively. Discount rates were not expected to have any significant impact on the results given the 12-month time horizon, and so were not considered.

### Base case model inputs

Clinical experts provided inputs for resource use items and their quantities, and the cost of an MRgFUS procedure. This expert clinical opinion was required to inform some model inputs given the lack of available data relevant to the Japanese healthcare setting from published sources based on the results of a literature review. Specifically, three neurosurgeons with extensive experience in the treatment of medication-refractory ET with both MRgFUS and unilateral DBS in the Japanese healthcare setting provided specific resource utilisation and cost inputs. Two of the clinicians validated each other’s inputs, and the third expert clinician adjudicated any disagreements. All other cost inputs for the model were derived from published sources as described below and in S1 and S2 Tables.

Pre-procedure resource use for both MRgFUS and unilateral DBS was assumed to be the same, comprising magnetic resonance imaging (MRI) and computed tomography (CT) scans and overnight hospitalisation costs. Pre-procedure costs were based on 2018 fee-for-service (FFS) tariffs, a payment system used by small hospitals in Japan based on costs derived from actual clinical practice in Japanese hospitals [31].

MRgFUS procedure costs included procedure fees, MRI use, local anaesthetic medication costs for the application of a stereotactic frame and hospitalisation costs. Unilateral DBS procedure costs included device costs, surgical fees, anaesthesia costs (delivery and medication), operative medication, CT imaging and hospitalisation costs. Costs for imaging and hospitalisation for both procedures, in addition to surgical fees and device costs and general anaesthesia for unilateral DBS, were obtained from the FFS tariffs [31], and both procedures were assumed to last four hours, as advised by the clinical experts. Drug costs for unilateral DBS were based on the 2018 National Drug Tariff in Japan [33].

As advised by the clinical experts, following the MRgFUS procedure, it was assumed an overnight hospital stay was required, with use of MRI the day after the procedure. For unilateral DBS, in the absence of available data in the Japanese healthcare setting, clinical experts advised that a hospital stay of eight days post-procedure would be required. Although length of hospital stay for DBS can be as low as two days in countries such as the United States [34], an analysis of Japanese health insurance claims from 2009–2015 for bilateral DBS found that the mean hospitalisation duration was 26–33 days [35], suggesting that the assumption of an eight day hospitalisation post-procedure for unilateral DBS applied may be an underestimate. Procedure costs following unilateral DBS also included one follow-up at-home educational session about device use within the first year. Post-procedure costs for both MRgFUS and unilateral DBS were based on 2018 FFS tariffs [31].

To address the potential for loss of efficacy with MRgFUS, the base case assumed that 8.9% of MRgFUS procedures would result in tremor recurrence within 12 months, and that 40% of simulated patients experiencing tremor recurrence with MRgFUS (i.e. 3.56% of all index MRgFUS procedures) would undergo a second procedure. These assumptions were previously accepted in health technology assessment of MRgFUS for medication-refractory ET in Canada [28], and validated by clinical experts as aligning with the use of MRgFUS in Japan. Japanese clinicians also advised that in Japan, RFT would likely be used in cases of tremor recurrence following MRgFUS. In line with the total costs for MRgFUS and unilateral DBS, the total cost for RFT was assumed to consist of pre-procedure (imaging and hospitalisation), procedure (anaesthesia, surgical fees and device costs) and post-procedure (hospitalisation) costs, based on FFS tariffs and the 2018 National Drug Tariff [31, 33].

In addition, extraction surgery to remove DBS electrodes was assumed to occur in 1% of unilateral DBS procedures per year, either due to breakage of equipment or an infection caused by the implant [36, 37]. The resource use for this removal procedure was assumed to comprise the costs of the surgical fee and anaesthesia delivery, taken from the 2018 FFS tariffs, and hospitalisation for 14 days, based on the input of clinical experts [31]. The cost of hospitalisation was also based on the 2018 FFS tariffs [31]. Clinical experts also advised that the duration of extraction surgery would be two hours.

The model inputs for the base case analysis of the model are summarised in S1 Table.

### Scenario analyses

#### Alternative use of DPC tariffs

FFS tariffs are only used in small hospitals in Japan, whereas larger hospitals are reimbursed according to Diagnosis Procedure Combination (DPC) tariffs, a diagnosis-related group (DRG)-based fixed-payment system used in large hospitals in Japan [32]. Therefore, a scenario analysis was performed to determine how the consideration of 2018 DPC tariffs would affect the total costs of MRgFUS and unilateral DBS.

In this DPC cost scenario, DPC tariffs replaced certain FFS tariffs used in the base case for pre-procedure and post-procedure costs, as well as hospitalisation fees for the day of the procedure [31, 32]. Model inputs for the scenario analysis using 2018 DPC tariffs are summarised in S2 Table.

#### Additional labour costs

Hospitalisation costs based on FFS or DPC tariffs include routine healthcare professional (HCP) labour costs associated with inpatient hospital stays [31, 32]. However, the Japanese Health Insurance Federation for Surgery (JHIFS; Gaihoren) suggests that these current tariffs may not accurately reflect the actual labour time incurred in many procedures, and as such has published hourly rates for labour costs (JHIFS tariffs) to account for costs that may not already be included in FFS or DPC tariffs [38]. Therefore, further scenario analyses were performed that included these additional HCP labour costs for MRgFUS and unilateral DBS procedures, to better reflect the actual cost of these procedures to hospitals. Labour costs were also added to the total cost of subsequent RFT procedures.

These scenario analyses were conducted using the most recent 2018 JHIFS tariff unit costs, or those from 2016 [38, 39]. The latter analysis was performed in order to evaluate the impact of substantial changes in the hourly rates of particular specialists between 2016 and 2018. Labour resource assumptions were based on the experience of clinical experts in Japan. Labour costs were then calculated from the hourly rates per professional, the number of staff needed and the duration of labour. Given the one-sided nature of labour costs associated with unilateral DBS extraction and device management, and the fact that the contributions from these activities to the total costs are very low, additional labour costs for these aspects of unilateral DBS were not considered.

A comparison of the JHIFS tariff for functional stereotaxic surgery, which excludes fees for device labour and costs, to the FFS-based Japanese medical care fee schedule found that the JHIFS tariff overestimated procedure costs by 40% [31, 38]. Therefore, when using JHIFS tariffs for model inputs concerning labour costs on the day of procedure, a correction factor of 0.7 was applied to account for this overestimation. Although pre-procedure costs were also based on JHIFS tariffs, these were only applicable to MRgFUS; hence, a conservative approach was adopted whereby the correction factor was not applied to pre-procedure costs in any analyses. Labour cost model inputs are summarised in S3 Table.

### Sensitivity analyses

One-way sensitivity analyses of the base case and DPC cost scenario analyses were performed by varying the proportion of MRgFUS procedures requiring a subsequent RFT procedure, the unilateral DBS extraction rate and the duration of hospitalisation for the unilateral DBS procedure; one-way sensitivity analyses varying the procedure time for MRgFUS were also performed for the 2018 labour cost scenario analyses using either base case or DPC cost scenario inputs (S4 Table). The unilateral DBS extraction rate, the duration of hospitalisation for the unilateral DBS procedure and the procedure time for MRgFUS were varied as these inputs were considered likely to differ across procedures based on feedback from expert clinicians in Japan. The proportion of MRgFUS procedures requiring a subsequent RFT procedure was varied to account for the uncertainty in the value assumed in the base case. These sensitivity analyses were conducted to assess the impact of variability or uncertainty in these parameters on the model results.

To further address the impact of uncertainty in the model parameters, a probabilistic sensitivity analysis (PSA) was also performed using a Monte Carlo simulation with 1,000 iterations to determine how simultaneously randomly sampling values for the base case parameters from pre-specified probabilistic distributions affected the base case results. Cost and quantity parameters were sampled from the gamma distribution, whilst percentage inputs were sampled from the beta distribution. In the absence of reported standard deviations in all cases, the distribution standard deviations were assumed to be 20% of the mean (base case) value. The quantities for premiums for activities were assumed to be the same as the frequencies for the relevant activities. It was also assumed that all simulated patients underwent a single primary procedure in hospital, however the associated hospitalisation and procedure fee costs were included in the PSA. A two-tailed student’s t-test at the 1% significance level was performed to determine if there was a statistically significant difference between the mean procedure costs for MRgFUS and DBS, after confirming the variance in the samples were the same using one-way ANOVA.

## Results

### Base case analysis

In the base case analysis of the cost-minimisation model, the total costs for MRgFUS and unilateral DBS were JPY2,145,037 and JPY2,545,417 per procedure, respectively. MRgFUS was therefore less costly than unilateral DBS by JPY400,380 per procedure. The procedure costs and post-procedure costs for MRgFUS were JPY278,393 and JPY121,987 lower than those for unilateral DBS, respectively, whilst the pre-procedure costs were identical (Table 1). Thus, the majority of cost savings achieved with MRgFUS versus unilateral DBS were procedure-related.

**Table 1:** Results of the base case analysis.

	Base case analysis (JPY)			
	Pre-procedure cost	Procedure cost	Post-procedure cost	Total
MRgFUS	50,610	2,032,440	61,987	**2,145,037**
Unilateral DBS	50,610	2,310,833	183,974	**2,545,417**
**Difference (MRgFUS versus DBS)**	**0**	**-278,393**	**-121,987**	**-400,380**

Difference and total values are reported to the nearest integer. **Abbreviations:** DBS: deep brain stimulation; JPY: Japanese Yen; MRgFUS: magnetic resonance-guided focussed ultrasound.

### Scenario analyses

In the DPC cost scenario, which replaced the majority of the FFS tariffs used in the base case with DPC tariffs, MRgFUS was also less costly than unilateral DBS by a similar magnitude (JPY414,691). Procedure and post-procedure savings with MRgFUS were well aligned with the base case analysis (JPY281,033 and JPY133,658 respectively) and again, the majority of cost savings were accrued during the procedure (Table 2).

**Table 2:** Costs per procedure by procedure stage in the DPC cost scenario.

	DPC cost scenario (JPY)			
	Pre-procedure cost	Procedure cost	Post-procedure cost	Total
MRgFUS	25,950	2,026,140	52,056	**2,104,146**
Unilateral DBS	25,950	2,307,173	185,714	**2,518,837**
**Difference (MRgFUS versus unilateral DBS)**	**0**	**-281,033**	**-133,658**	**-414,691**

Difference and total values are reported to the nearest integer. **Abbreviations**: DBS: deep brain stimulation; JPY: Japanese Yen; MRgFUS: magnetic resonance-guided focussed ultrasound.

The cost differences between MRgFUS and unilateral DBS observed in both the base case and the DPC cost scenario were increased when additional HCP labour costs were included (Table 3). Compared to the base case without additional HCP labour costs, cost savings from MRgFUS increased to JPY736,143 and JPY515,910 when adding 2018 labour costs or 2016 labour costs, respectively.

**Table 3:** Overall costs per procedure in the labour cost scenario analyses.

	Base case	Labour cost (2016)^a^	Labour cost (2018)^a^	Total with labour cost (2016)^a^	Total with labour cost (2018)^a^
**Base case analysis**					
MRgFUS	2,145,037	871,840	930,977	**3,016,877**	**3,076,014**
Unilateral DBS	2,545,417	987,370	1,266,740	**3,532,787**	**3,812,157**
**Difference (MRgFUS versus unilateral DBS)**	**-400,380**	**-115,530**	**-335,763**	**-515,910**	**-736,143**
**DPC cost scenario**					
MRgFUS	2,104,146	871,840	930,977	**2,975,986**	**3,035,123**
Unilateral DBS	2,518,837	987,370	1,266,740	**3,506,207**	**3,785,577**
**Difference (MRgFUS versus unilateral DBS)**	**-414,691**	**-115,530**	**-335,763**	**-530,221**	**-750,454**

Difference and total values are reported to the nearest integer.

^a^2018 JHIFS labour costs on the day of procedure were adjusted using a multiplication factor of 0.7 to account for the overestimation of these costs when using the JHIFS tariff compared to FFS tariffs.

**Abbreviations:** DBS: deep brain stimulation; JPY: Japanese Yen; MRgFUS: magnetic resonance-guided focussed ultrasound; RFT: radiofrequency thalamotomy.

### One-way sensitivity analyses

The proportion of MRgFUS procedures requiring a subsequent RFT procedure, the unilateral DBS extraction rate and the duration of hospitalisation for the unilateral DBS procedure were varied in one-way sensitivity analyses of the base case and DPC cost scenario analyses; a one-way sensitivity analysis varying procedure time for MRgFUS was performed for the 2018 labour cost scenario analyses using either base case or DPC cost inputs (Table 4). In all cases, MRgFUS remained cost saving versus unilateral DBS, demonstrating the robustness of this result. Varying either the proportion of MRgFUS procedures requiring a subsequent RFT procedure or the unilateral DBS extraction rate in the base case and DPC cost scenario analyses had a marginal impact on the cost savings achieved by MRgFUS. In contrast, in the 2018 labour cost scenario analysis using base case inputs, reducing the MRgFUS procedure length from four to two hours increased the cost savings for MRgFUS versus unilateral DBS from JPY736,143 to JPY1,150,137; increasing the procedure length of MRgFUS from four to six hours reduced cost savings to JPY322,149. Although reducing the post-procedure hospitalisation duration for unilateral DBS from eight to two days lowered the cost savings for MRgFUS (from JPY400,380 to JPY277,290 using base case inputs), MRgFUS remained cheaper than unilateral DBS.

**Table 4:** Overall costs per procedure for MRgFUS and unilateral DBS (sensitivity analyses).

	Analyses without labour costs							Analyses with labour costs		
	Base case	Proportion of MRgFUS procedures requiring subsequent RFT procedure		Unilateral DBS extraction rate		Unilateral DBS post-procedure hospitalisation duration		Scenario analysis with 2018 labour costs added to the base case^a^	MRgFUS procedure duration (using 2018 labour costs)^a^	
		0%	10%	0%	2%	2 days	10 days		2 hours	6 hours
**Base case analysis**										
MRgFUS	2,145,037	2,115,300	2,198,830	2,145,037	2,145,037	2,145,037	2,145,037	3,086,014	2,662,020	3,490,008
Unilateral DBS	2,545,417	2,545,417	2,545,417	2,540,223	2,550,611	2,442,957	2,586,237	3,812,157	3,812,157	3,812,157
** Difference (MRgFUS versus unilateral DBS)**	**-400,380**	**-430,117**	**-346,587**	**-395,186**	**-405,574**	**-277,920**	**-441,200**	**-736,143**	**-1,150,137**	**-322,149**
**DPC cost scenario**										
MRgFUS	2,104,146	2,078,040	2,151,371	2,104,146	2,104,146	2,104,146	2,104,146	3,035,123	2,621,129	3,449,117
Unilateral DBS	2,518,837	2,518,837	2,518,837	2,513,643	2,524,031	2,390,217	2,557,197	3,785,577	3,785,577	3,785,577
** Difference (MRgFUS versus unilateral DBS)**	**-414,691**	**-440,797**	**-367,466**	**-409,497**	**-419,885**	**-286,071**	**-453,051**	**-750,434**	**-1,164,448**	**-336,460**

Difference and total values are reported to the nearest integer.

^**a**^2018 JHIFS labour costs on the day of procedure were adjusted using a multiplication factor of 0.7 to account for the overestimation of these costs when using the JHIFS tariff compared to FFS tariffs.

**Abbreviations:** DBS: deep brain stimulation; JPY: Japanese Yen; MRgFUS: magnetic resonance-guided focussed ultrasound; RFT: radiofrequency thalamotomy.

### Probabilistic results

**Fig 1:**
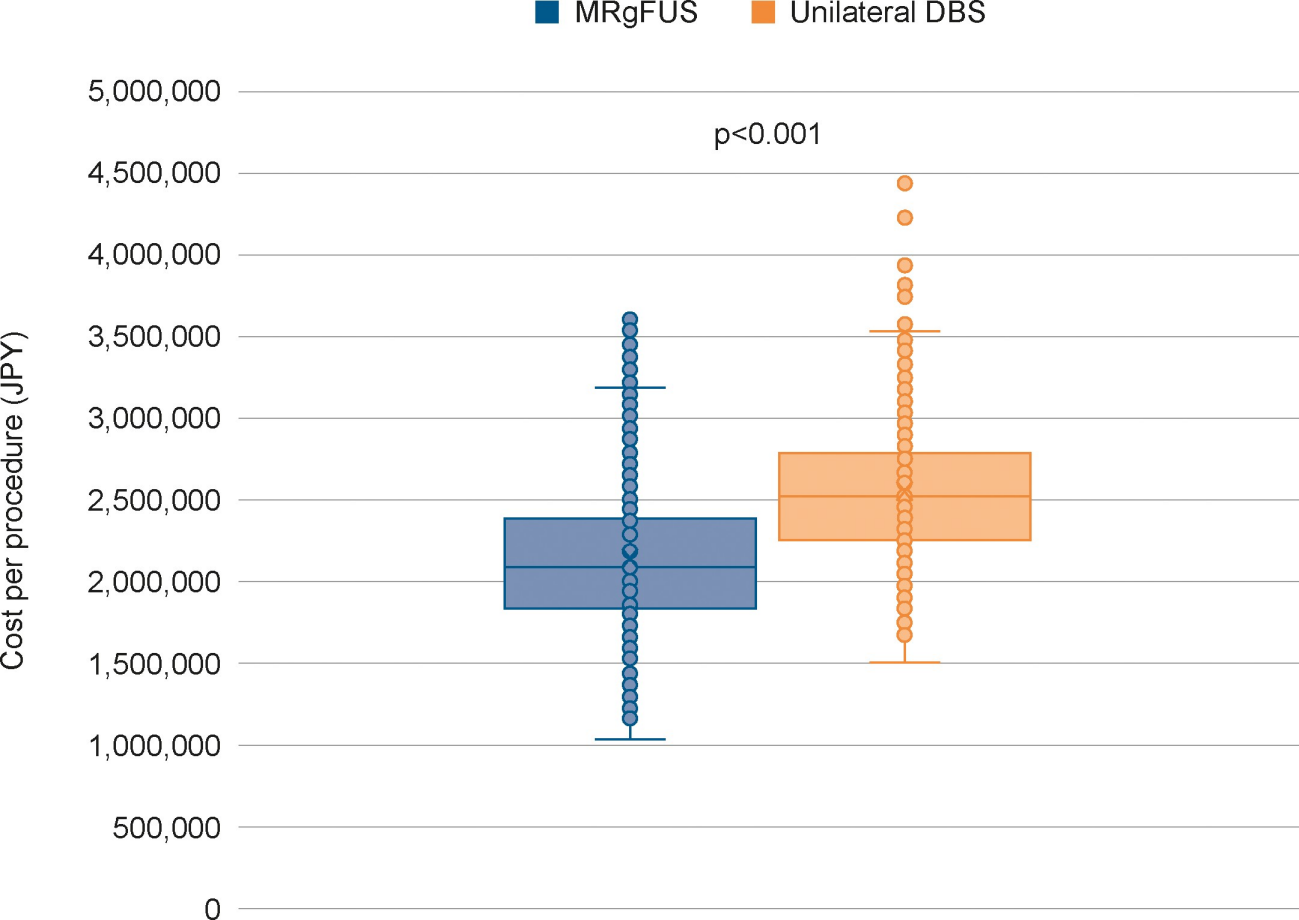
Results of PSA. Box and whisker plot presenting median per procedure costs for MRgFUS and unilateral DBS. The boxes represent the 1^st^ and 3^rd^ quartiles (interquartile range); lower error bars indicate up to 1.5 times the interquartile range below the first quartile, and upper error bars indicate up to 1.5 times the interquartile range above the third quartile.

Based on the PSA, the probabilistic estimates for MRgFUS and unilateral DBS were JPY2,143,337 and JPY2,546,196 per procedure, respectively. MRgFUS achieved cost savings of JPY402,859 and was less costly than unilateral DBS in 78.5% of iterations. Overall, MRgFUS was significantly cheaper than DBS (p<0.001). The results of the PSA are presented in Fig 1.

## Discussion

### Main findings

This study represents the first cost-minimisation model comparing MRgFUS with unilateral DBS. The results demonstrate that MRgFUS was cost saving when compared with unilateral DBS up to 12 months post-procedure in the base case of the model, with cost savings also observed when some FFS tariffs were replaced with DPC tariffs in a scenario analysis. Therefore, the results are equally robust when evaluated in both small or large institutions. When labour costs were included in further scenario analyses, the savings associated with MRgFUS versus unilateral DBS were even greater than those in the base case, demonstrating that unilateral DBS requires greater utilisation of HCP resource. Furthermore, cost savings were maintained in all sensitivity analyses, which explored the effects of varying the proportion of MRgFUS procedures requiring a subsequent RFT procedure, the unilateral DBS extraction rate, the unilateral DBS hospitalisation duration and the MRgFUS procedure duration. The PSA result indicates that this analysis presents a robust evaluation of the cost differences between MRgFUS and DBS. Overall, the cost savings achieved with MRgFUS versus unilateral DBS were found to be highly robust.

The majority of cost savings achieved with MRgFUS versus unilateral DBS in the model were procedure-related, which is consistent with the minimally invasive nature of MRgFUS compared to DBS. Specifically, a large part of the cost difference between the two procedures is derived from the need for both general anaesthesia and additional medications during unilateral DBS compared with the need for only local anaesthesia for MRgFUS. Cost savings were also accrued post-procedure, in line with the shorter recovery period for MRgFUS compared to unilateral DBS and the avoidance of subsequent procedures needed due to breakage of equipment or infection [23, 36, 37].

Overall, the estimated cost savings for MRgFUS versus unilateral DBS could be considered conservative. With a time horizon of 12 months, it is likely that patients in clinical practice would require multiple follow-up visits for the examination and adjustment of unilateral DBS, yet this model included just one for the patients in the simulation. As this cost is unique to unilateral DBS, this simplification would likely underestimate the cost savings achieved with MRgFUS. Furthermore, although the cost of DBS extraction (due to infection or hardware failure, for example) was considered in the model, treatment costs for AEs associated with each procedure were otherwise not included due to the lack of head-to-head data comparing AEs for MRgFUS versus unilateral DBS. AE costs would also be expected to have a greater impact on overall costs if the model time horizon were extended. Procedure-related serious AEs for MRgFUS have been found to be very infrequent (1.6%), with no cases of intracerebral haemorrhages or infections reported in an analysis of five MRgFUS studies, as patients avoid AEs associated with invasive techniques [26, 30]. In comparison, studies of unilateral DBS report intracranial haemorrhage rates of 1% and wound infections in 3–6% of patients at 6–36 months follow-up [18, 40]. Movement-related AEs may also be less frequent with MRgFUS versus unilateral DBS. In a study comparing MRgFUS to unilateral DBS, gait disturbance was reported in 33% and 85% patients and dysarthria in 7% and 8% patients undergoing MRgFUS and DBS, respectively, at three months post-procedure [15]. Therefore, the costs associated with managing AEs for MRgFUS would be expected to be lower than those for unilateral DBS, due to a lower incidence. Additionally, the ongoing device-related costs associated with DBS, such as battery replacement every two to five years, would be expected to increase the potential cost savings of MRgFUS in the long-term [41].

Few economic evaluations of MRgFUS versus DBS in medication-refractory ET have previously been performed in different healthcare settings, although those that have been conducted–in Sweden, the United States and Canada–have shown consistently favourable results for MRgFUS, both when considering procedure costs only and longer-term follow-up [27–29]. A Swedish study found that the cost per patient using MRgFUS was over three times lower compared to DBS (SEK48,000 versus SEK170,000), even before the costs of DBS pulse generator replacement surgery were considered (SEK86,000 per replacement, required every four to five years). However, it is unclear whether this analysis considered unilateral and/or bilateral DBS [27]. In Canada, the total cost of MRgFUS was less than half of that calculated for DBS (when considering the costs of primary surgery, monitoring, medications, reoperation and managing AEs for both procedures and battery replacement for DBS only), with costs of CAD23,507 and CAD57,535 for MRgFUS and DBS, respectively. In this analysis, it was assumed that 90% of DBS procedures were performed unilaterally, respectively [28]. In the United States, MRgFUS was associated with a cost saving of USD8,278 per procedure compared with DBS, with evidence from DBS studies based on reports in which the majority (more than 60%) of patients received a unilateral procedure [29]. Overall, these previous economic studies performed globally appear to align with the present economic model, demonstrating cost savings achieved with MRgFUS compared to DBS.

### Study limitations

It is important to acknowledge that this model may have limitations in estimating the true cost difference between MRgFUS and unilateral DBS in clinical practice. The cost-minimisation approach assumes MRgFUS and unilateral DBS result in equal tremor improvements throughout the time horizon of the model. This assumption was considered reasonable given that a small retrospective study and a recently published ITC found no evidence of a difference in efficacy up to 12 months post-procedure [15, 30]. In order to address uncertainty in the assumption of equal efficacy, the model captures costs associated with loss of efficacy with MRgFUS by assuming that a proportion of procedures require a subsequent RFT procedure due to tremor recurrence. It is acknowledged that a longer time horizon may be preferable for economic modelling, however, there is insufficient data comparing MRgFUS and unilateral DBS to extend the model time horizon. Importantly though, the pivotal RCT investigating MRgFUS in ET has evidence to suggest that tremor reduction is maintained up to four years post-procedure [25]. It was also not possible to include AEs in the model without introducing further uncertainty into the model results as no analyses have been performed directly comparing AEs for MRgFUS versus unilateral DBS. However, as described above, the exclusion of AEs likely underestimates the cost savings achieved with MRgFUS versus unilateral DBS. Overall, the field would benefit from future research directly comparing these two techniques in terms of efficacy and safety over a >12 month period of follow-up to enable economic modelling over a longer time horizon.

Secondly, the DPC tariff used as a resource input in the DPC cost scenario is based on medical fee estimations that may not fully reflect the cost to hospitals [32]. Further analysis using hospital-based resource use and cost data in place of this tariff could provide an estimate of the cost savings that may be achieved with MRgFUS versus unilateral DBS that better reflects clinical practice in Japan. In addition, the impact of using MRI scanners for MRgFUS procedures on throughput for other MRI-dependent services was not included as this was outside the perspective of the model. However, this would be expected to be an important factor for decision-makers in healthcare facilities.

Finally, this model compared MRgFUS to unilateral implantation of DBS only. Therefore, bilateral implantation of DBS may still be an appropriate choice for patients who would be best treated with such a procedure in clinical practice; however, bilateral DBS was not considered within the scope of this model.

### Conclusions

The cost-minimisation model presented here indicates that MRgFUS is cost saving compared with unilateral DBS for the treatment of medication-refractory ET in the Japanese healthcare setting. This conclusion will be useful to support decision making when selecting the procedure with the most favourable cost profile in the treatment of medication-refractory ET in clinical practice in Japan, without compromising on clinical or patient outcomes. Healthcare payers may achieve healthcare cost savings through the replacement of unilateral DBS by MRgFUS, due to the associated decrease in cost per procedure. Total cost savings may also increase further over time as the number of ET patients in clinical practice is expected to rise in the future, reflecting an increase in the proportion of the Japanese population that is aged >65 years [42]. If MRgFUS becomes more widely accessible as a result of its positive economic profile, more patients could benefit from its non-invasive nature, decreased incidence of AEs, the avoidance of extraction or revision operations and a shorter recovery period of hospitalisation [9, 15, 18, 26, 40, 43].

## Supporting information

S1 Table Model inputs for the base case analysis. (DOCX)

S2 Table Model inputs for the scenario analysis using DPC tariffs. (DOCX)

S3 Table Labour costs included in the scenario analyses. (DOCX)

S4 Table Parameters varied in the sensitivity analyses. (DOCX)
