# Supplementary material for: Cost-minimisation model of magnetic resonance-guided focussed ultrasound therapy compared to unilateral deep brain stimulation for essential tremor treatment in Japan
Source: PLoS One. 2019 Jul 17;14(7):e0219929. doi: 10.1371/journal.pone.0219929 (PMC6636755; doi:10.1371/journal.pone.0219929)
Supplement: S1 Table — (DOCX) [file pone.0219929.s001.docx]

Supporting Information

**S1 Table. Model inputs for the base case analysis**

| **Parameter** | **Unit cost (JPY)** | **Quantity** | **Total  (JPY)** | **Unit cost source** |
| --- | --- | --- | --- | --- |
| **Pre-procedure** | | | | |
| MRgFUS and unilateral DBS | | | | |
| *Imaging* | | | | |
| E202 MRI scan (1.5 to 3.0 tesla)* | 13,300 | 1 | 13,300 | 2018 FFS tariff [31] |
| E000 Premium for computer management of MRI imaging* | 1,200 | 1 | 1,200 |  |
| E200 CT scan (multi-slice CT over 64 slices)*^a^ | 10,000 | 1 | 10,000 |  |
| E000 Premium for computer management of CT imaging* | 1,200 | 1 | 1,200 |  |
| E203 Diagnosis fee for CT/MRI*^b^ | 4,500 | 1 | 4,500 |  |
| *Hospitalisation* | | | | |
| A100-1 Basic fee for hospitalisation (general ward, one nurse per seven in-hospital patients)* | 15,910 | 1 | 15,910 | 2018 FFS tariff [31] |
| A100-1 Premium for early discharge (within 14 days)* | 4,500 | 1 | 4,500 |  |
| **Procedure** | | | | |
| MRgFUS | | | | |
| *Procedure fee* | | | | |
| Procedure fee for MRgFUS | 2,000,000 | 1 | 2,000,000 | Validated input from expert clinicians in Japan |
| *Imaging* | | | | |
| E202 MRI scan (1.5 to 3.0 tesla) (2^nd^ time or later in a month)^a^ | 10,640 | 1 | 10,640 | 2018 FFS tariff [31] |
| E000 Premium for computer management of MRI imaging | 1,200 | 1 | 1,200 |  |
| *Local anaesthesia* | | | | |
| Xylocaine 0.5% for I.M. 3 mL (local anaesthesia) | 95 | 2 | 190 | National Drug Tariff (2018) [33] |
| *Hospitalisation* | | | | |
| A100-1 Basic fee for hospitalisation (general ward, one nurse per seven in-hospital patients)* | 15,910 | 1 | 15,910 | 2018 FFS tariff [31] |
| A100-1 Premium for early discharge (within 14 days)* | 4,500 | 1 | 4,500 |  |
| Unilateral DBS | | | | |
| *Surgical fee and device costs* | | | | |
| K181 Surgery for implanting device into the brain (unilateral) | 651,000 | 1 | 651,000 | 2018 FFS tariff [31] |
| K930 Premium for measuring spinal cord-evoked potential | 31,300 | 1 | 31,300 |  |
| 085 Leads for deep brain stimulation (four electrodes) | 140,000 | 1 | 140,000 |  |
| 086 Lead for brain and spinal stimulation (adapter) | 34,800 | 1 | 34,800 |  |
| 087 Implantable devices for brain and spinal stimulation (for essential tremor) (four electrodes) | 1,240,000 | 1 | 1,240,000 |  |
| 088 Intracranial electrodes for electroencephalogram measurement | 36,500 | 2 | 73,000 |  |
| 039 Disposable catheters for indwelling the bladder (standard type) | 1,620 | 1 | 1,620 |  |
| *Imaging* | | | | |
| E200 CT scan (multi-slice CT over 64 slices) (2^nd^ time or later in a month)^a^ | 8,000 | 1 | 8,000 | 2018 FFS tariff [31] |
| E000 Premium for computer management of CT imaging | 1,200 | 1 | 1,200 |  |
| *General anaesthesia* | | | | |
| L008 Closed-loop general anaesthesia by endotracheal intubation or masks (<2 hours) | 60,000 | 1 | 60,000 | 2018 FFS tariff [31] |
| Extra fee per 30 min. for anaesthesia >2 hours (for two hours) | 6,000 | 4 | 24,000 |  |
| L009 Premium for anaesthesia management | 10,500 | 1 | 10,500 |  |
| Oxygen (unit: L) | 0.19 | 1,430^c^ | 271.7 | National Drug Tariff (2018) [33] |
| Xylocaine injection syringe 1% 10 mL | 201 | 1 | 201 |  |
| 1% Diprivan injection-kit 500mg 50 mL | 1,754 | 1 | 1,754 |  |
| Popscaine 0.25% inj. syringe 2.5 mg/10 mL | 428 | 2 | 856 |  |
| Popscaine 0.5% inj. syringe 50 mg/10 mL | 599 | 1 | 599 |  |
| *Additional medication received during unilateral DBS procedure* | | | | |
| Medication^d^ | 11,321 | 1 | 11,321 | National Drug Tariff (2018) [33] |
| *Hospitalisation* | | | | |
| A100-1 Basic fee for hospitalisation (general ward, one nurse per seven in-hospital patients)* | 15,910 | 1 | 15,910 | 2018 FFS tariff [31] |
| A100-1 Premium for early discharge (within 14 days)* | 4,500 | 1 | 4,500 |  |
| **Post-procedure** | | | | |
| MRgFUS | | | | |
| *Imaging and hospitalisation* | | | | |
| E202 MRI scan (1.5 to 3.0 tesla) (2^nd^ time or later in a month)*^a^ | 10,640 | 1 | 10,640 | 2018 FFS tariff [31] |
| E000 Premium for computer management of MRI imaging* | 1,200 | 1 | 1,200 |  |
| A100-1 Basic fee for hospitalisation (general ward, one nurse per seven in-hospital patients)* | 15,910 | 1 | 15,910 | 2018 FFS tariff [31] |
| A100-1 Premium for early discharge (within 14 days)* | 4,500 | 1 | 4,500 |  |
| *RFT following tremor recurrence after MRgFUS* | | | | |
| Percentage of MRgFUS procedures requiring subsequent RFT | 3.56% | | | Health Quality Ontario [28] |
| RFT total cost* | 835,301 | 1 | 835,301 | 2018 FFS tariff [31]  National Drug Tariff (2018) [33] |
| Unilateral DBS | | | | |
| *Hospitalisation and outpatient treatment* | | | | |
| A100-1 Basic fee for hospitalisation (general ward, one nurse per seven in-hospital patients)* | 15,910 | 8 | 127,280 | 2018 FFS tariff [31] |
| A100-1 Premium for early discharge (within 14 days)* | 4,500 | 8 | 36,000 |  |
| C110-2 Home treatment tremor stimulation device treatment instruction guidance management fee* | 8,100 | 1 | 8,100 |  |
| Premium for introductory phase for at-home education (initial three months after procedure)* | 1,400 | 1 | 1,400 |  |
| C167 Premium for transmitter management*^e^ | 6,000 | 1 | 6,000 |  |
| *Unilateral DBS extraction* | | | | |
| Percentage of unilateral DBS simulated patients requiring extraction | 1% | | | Falowski *et al.* (2016) [36]  Binder *et al.* (2005) [37] |
| K181-3 Intracranial electrode extraction operation | 128,800 | 1 | 128,800 | 2018 FFS tariff [31] |
| L008 Closed-loop general anaesthesia by endotracheal intubation or masks (<2 hours) | 60,000 | 1 | 60,000 |  |
| L009 Premium for anaesthesia management | 10,500 | 1 | 10,500 |  |
| Oxygen (unit: L)^f^ | 0.19 | 715^c^ | 135.9 | National Drug Tariff (2018) [33] |
| Medication^f^ | 7,366 | 1 | 7,366 |  |
| Antibiotics^g^ | 1,919 | 14 | 26,866 |  |
| A100-1 Basic fee for hospitalisation (general ward, one nurse per seven in-hospital patients) | 15,910 | 14 | 222,740 | 2018 FFS tariff [31] |
| A100-1 Premium for early discharge (within 14 days) | 4,500 | 14 | 63,000 |  |

*Indicates an input that has an alternative value in the scenario analysis using 2018 DPC tariff costs.

^a^The cost of imaging for the 2^nd^ time or later in a month was 80% of the cost for the first imaging procedure, defined by the FFS tariff [[31](#_ENREF_31)]. ^b^This fee was charged only once a month regardless of the number or types of imaging procedure received. ^c^This value included multiplication by a correction factor of 1.3 to account for wastage. ^d^Consisting of 1x normal saline 500mL (manufacturer: Otsuka); 1x normal saline 100mL (manufacturer: Otsuka); 1x Veen-F injection 500mL; 1x Solmalt infusion 500mL; 1x Replas 3 injection 500mL; 2x Cefotiam hydrochloride for I.V. infusion; 1x Tranexamic acid injection 1g 10% 10mL; 1x Bridion 200mg 2mL; 1x Eslax I.V. 50mg/5.0mL 5mL; 1x Atropine sulfate injection 0.5mg "Fuso" 0.05m% 1mL. ^e^This fee was charged for simulated patients undergoing at-home tremor management with unilateral DBS using a special transmitter. ^f^Considering the duration of unilateral DBS extraction surgery, it was assumed that half the amount of oxygen and medication were used as unilateral DBS implant surgery. ^g^Consisting of Vancomycin Hydrochloride for Injection 1 g (manufacturer: Pfizer) and Vancomycin Hydrochloride for Injection 0.5 g (manufacturer: Pfizer).

**Abbreviations:** CT: computerised tomography; DBS: deep brain stimulation; FFS: fee-for-service; I.M.: intramuscular; I.V.: intravenous; JPY: Japanese Yen; MRgFUS: magnetic resonance-guided focussed ultrasound; MRI: magnetic resonance imaging; RFT: radiofrequency thalamotomy.
