# Supplementary material for: Cost-minimisation model of magnetic resonance-guided focussed ultrasound therapy compared to unilateral deep brain stimulation for essential tremor treatment in Japan
Source: PLoS One. 2019 Jul 17;14(7):e0219929. doi: 10.1371/journal.pone.0219929 (PMC6636755; doi:10.1371/journal.pone.0219929)
Supplement: S3 Table — (DOCX) [file pone.0219929.s003.docx]

Supporting Information

**S3 Table. Labour costs included in the scenario analyses**

| **HCP** |  | **MRgFUS** | | | **Unilateral DBS** | | | **RFT^b^** |
| --- | --- | --- | --- | --- | --- | --- | --- | --- |
|  | **Cost per hour (JHIFS tariff; JPY)** | **Number of staff needed** | **Duration (hours)** | **Total (JPY)^a^** | **Number of staff needed** | **Duration (hours)** | **Total (JPY)^a^** |  |
| **Pre-procedure** | | | | | | | |  |
| *Physician (Technical level D)* | 2018: 68,080  2016: 17,400 | 1 | 1 | 2018: 68,080  2016: 17,400 | 0 | N/A | 0 |  |
| *MRI technician* | 2018: 2,760  2016: 2,650 | 1 | 2 | 2018: 5,520  2016: 5,300 | 0 | N/A | 0 |  |
| **Procedure^a^** | | | | | | | |  |
| *Operating surgeon (Technical level E)* | 2018: 138,450  2016: 139,110 | 1 | 4 | 2018: 387,660  2016: 389,508 | 1 | 4 | 2018: 387,660  2016: 389,508 |  |
| *Cooperative physician (Technical level D)* | 2018: 92,300  2016: 92,740 | 1 | 4 | 2018: 258,440  2016: 259,672 | 1 | 4 | 2018: 258,440  2016: 259,672 |  |
| *Cooperative physician (Technical level C)* | 2018: 56,460  2016: 56,630 | 1 | 4 | 2018: 158,088  2016: 158,564 | 1 | 4 | 2018: 158,088  2016: 158,564 |  |
| *Nurse* | 2018: 2,980  2016: 2,840 | 1 | 4 | 2018: 8,344  2016: 7,952 | 2 | 4 | 2018: 16,688  2016: 15,904 |  |
| *Technician* | 2018: 2,760  2016: 2,650 | 2 | 4 | 2018: 15,456  2016: 14,840 | 2 | 4 | 2018: 15,456  2016: 14,840 |  |
| *Anaesthetist* | 2018: 56,460  2016: 28,315 | 0 | N/A | 0 | 1 | 4 | 2018: 158,088  2016: 79,282 |  |
| **Post-procedure** | | | | | | | |  |
| *Physician (Technical level D)* | 2018: 68,080  2016: 17,400 | 0 | N/A | 0 | 1 | 4^c^ | 2018: 272,320  2016: 69,600 |  |
| **TOTAL** | | 2018: 930,977  2016: 871,840 | | | 2018: 1,266,740  2016: 987,370 | | | 2018: 825,542^d^  2016: 522,584^d^ |

^a^2018 JHIFS labour costs on the day of procedure were adjusted using a multiplication factor of 0.7 to account for the overestimation of these costs when using the JHIFS tariff compared to FFS tariffs. ^b^Only considered for the proportion of MRgFUS procedures requiring a subsequent RFT procedure. ^c^0.5 hours over eight days. ^d^Assuming a two hour procedure with a hospital stay of five days post-procedure.

**Abbreviations:** DBS: deep brain stimulation; HCP: healthcare professional; JPY: Japanese Yen; MRgFUS: magnetic resonance-guided focussed ultrasound; N/A: not applicable; RFT: radiofrequency thalamotomy.
