# Supplementary material for: Cost-minimisation model of magnetic resonance-guided focussed ultrasound therapy compared to unilateral deep brain stimulation for essential tremor treatment in Japan
Source: PLoS One. 2019 Jul 17;14(7):e0219929. doi: 10.1371/journal.pone.0219929 (PMC6636755; doi:10.1371/journal.pone.0219929)
Supplement: S4 Table — (DOCX) [file pone.0219929.s004.docx]

Supporting Information

**S4 Table. Parameters varied in the sensitivity analyses**

|  | **Parameter** | **Assumption in base case and DPC cost scenario** | **Low assumption in one-way sensitivity analysis** | **High assumption in one-way sensitivity analysis** |
| --- | --- | --- | --- | --- |
| **Analyses without labour costs** | Proportion of MRgFUS procedures requiring subsequent RFT procedure | 3.56% | 0% | 10% |
|  | Extraction rate for unilateral DBS | 1% | 0% | 2% |
|  | Unilateral post-procedure DBS hospitalisation | 8 days | 7 days | 12 days |
| **Analyses with labour costs** | MRgFUS procedure duration^a^ | 4 hours | 2 hours | 6 hours |

^a^These sensitivity analyses used base case or DPC cost scenario inputs and 2018 JHIFS labour costs. 2018 JHIFS labour costs on the day of procedure were adjusted using a multiplication factor of 0.7 to account for the overestimation of these costs when using the JHIFS tariff compared to FFS tariffs.

**Abbreviations:** DBS: deep brain stimulation; MRgFUS: magnetic resonance-guided focussed ultrasound; RFT: radiofrequency thalamotomy.
